# Robot-assisted radical prostatectomy: a case series of the first 100 patients -constitutional introduction and implementation on the basis of comprehensive department of minimal invasive surgery center**-**

**DOI:** 10.1186/1756-0500-6-436

**Published:** 2013-10-30

**Authors:** Takehiro Sejima, Toshihiko Masago, Shuichi Morizane, Katsuya Hikita, Naoto Kobayashi, Akihisa Yao, Kuniyasu Muraoka, Masashi Honda, Hiroya Kitano, Atsushi Takenaka

**Affiliations:** 1 Division of Urology, Department of Surgery, Tottori University Faculty of Medicine, 36-1 Nishimachi, Yonago 683-8504, Japan; 2 Minimal invasive surgery center, Tottori University Faculty of Medicine, Yonago, Japan

**Keywords:** Robot-assisted radical prostatectomy, Minimum invasive surgery, Peri-operative complication, Oncological outcome, Functional outcome

## Abstract

**Background:**

Although a very small number of Japanese hospitals had been performing robotic surgery before 2011, the number now using it is increasing rapidly due to the application of health insurance to robotic surgery for prostate cancer (PCa) since April, 2012. We report our initial experience of treating 100 patients by robot-assisted radical prostatectomy (RARP) with a focus on constitutional introduction and implementation based on minimal invasive surgery center (MISC) and patient outcomes.

**Methods:**

The MISC involved all of the hospital sections related to robotic surgery including four surgery departments, anesthesiology, operating room nurses, medical engineers. The data were prospectively collected from the first 100 consecutive patients who underwent RARP under supervision of MISC for localized PCa from October 2010 to December 2012.

**Results:**

During the period of our initial 100 cases of RARP, the gynecology, respiratory and digestive surgery departments performed initial cases of 20, 33 and 23 robotic surgeries under control of MISC. Peri-operative complications in RARP appeared to be minimal with no cases of intra-operative open conversion. The positive surgical margin rate was 19% for the entire series. At the median follow-up time of 11.9 months, 91% of patients had undetectable PSA levels, and 76% of patients were not using pads. Sequential urinary functional data indicated a significant beneficial effect on lower urinary tract symptoms beyond cancer control over a period of several months. Although the pre-operative potent patient number was small, the transitions of constant potency recovery at precise time points were shown according to different nerve sparing procedures.

**Conclusions:**

This is the first report of an initial 100 RARP cases that were implemented using the constitutional framework of an academic institution. The MISC is providing immeasurable benefits from the aspects of patient safety and education for the robotic surgical team. RARP is a safe and efficient method for achieving PCa control together with functional preservation, even during the initial trial for this procedure.

## Background

Prostate cancer (PCa) has been the most common non-cutaneous malignancy in United States (US). men since 1984, now accounting for one quarter of all such cancers [1]. The Recent age adjusted incidence rate of PCa in Japan was 15.1/100,000 population which is a relatively low risk compared to 90 to 110/100,000 population rate in western countries because PCa incidence varies by race/ethnicity [2]. Although the specific cause of PCa initiation and progression are not yet known, considerable evidence suggests that both genetics and environment play a role in the origin and evolution of this disease. Throughout the history of definitive therapy for localized PCa, surgical therapy has played a central role in the trend toward minimal invasive technique. Robot-assisted radical prostatectomy (RARP) using the da Vinci surgical system started in 2000 [3], and has spread rapidly retaining the concept of minimal invasive surgery. Now, it has become the established surgical treatment for localized PCa in the US [4]. The magnified 3-D view, useful scissors with multi-joints, and the ease of the manipulation of the da Vinci system have provided surgeons with exceptional detail of the pelvic anatomy and made radical prostatectomy a minimally invasive surgery. As a result, RARP could be an epoch-making surgical procedure with which we could achieve the three competing goals of radical prostatectomy; that is, cancer control, urinary continence and erectile function [5].

Although RARP was introduced in 2006 in Japan, the spread of RARP has been very slow because of off-label application of Japanese health insurance at that time. However, the Japanese Ministry of Health, Labor and Welfare admitted the application of health insurance for RARP on April 2012; therefore, the rapid spread of RARP is expected. A concern with regard to the rapid spread of a new surgical innovation is the issue of patient safety. Reduced patient safety occurs due to insufficient preparation and inadequate surgical technique during the introduction of a new technology. To eliminate such concerns, the introduction and implementation of robotic surgeries in our institution were controlled by the minimal invasive surgery center (MISC), which runs robotic surgeries comprehensively not only in urology but also in other surgical departments. In this study, we report our initial experience of 100 patients treated by RARP with a focus on constitutional introduction and implementation based on a MISC and patient outcomes based on the concept of pentafecta [6].

## Methods

### Introduction and implementation of robotic surgery -Comprehensive management by a MISC-

**Figure 1:**
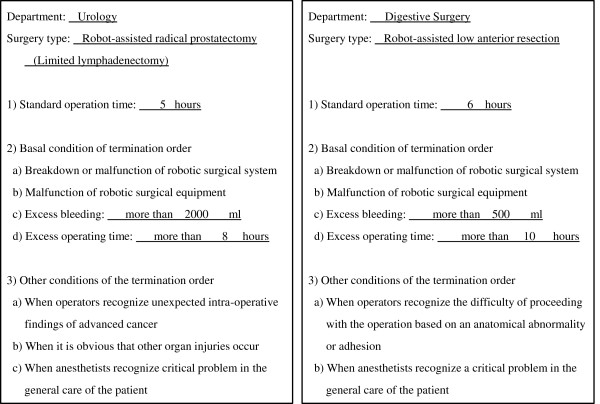
**The termination order (English version) for RARP and low anterior resection are shown.** The original documents are described in Japanese.

**Figure 2:**
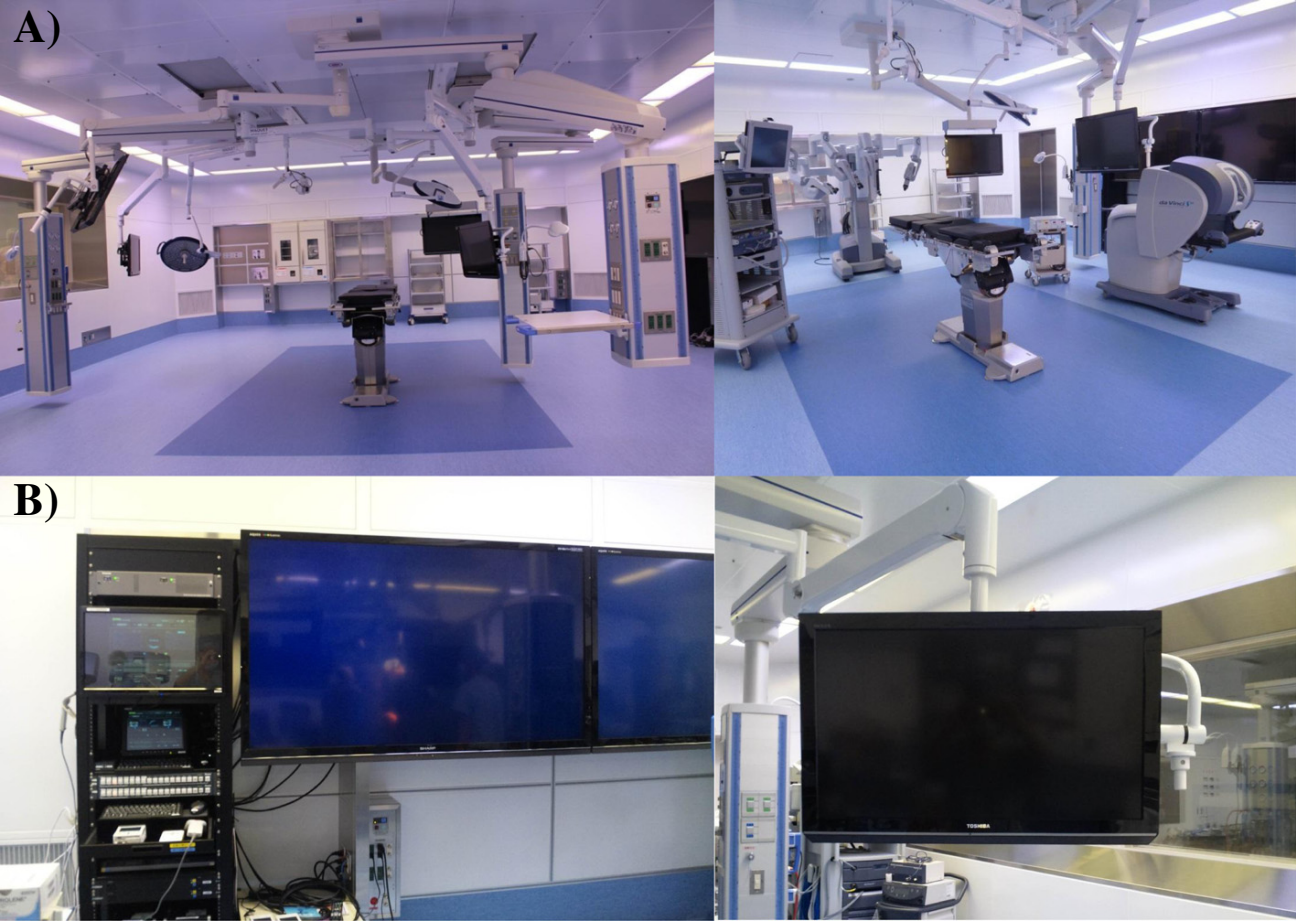
**The inside views and equipment of the MISC robotic-operating theater are shown. A)** The floor space is 98 m^2^. **B)** This theater has six fixed monitors and one movable monitor. The six fixed monitors include two sets of 70 inch and four sets of 32 inch-sized monitors. One movable monitor is 46 inch in size. Four sets of 32 inch size fixed monitors and one movable monitor are capable of 3D display.

Our institution purchased the daVinci S robot on August, 2010 in the order of eighth in Japan. Two months later, the first RARP case was performed by the chairman of urology (A.T.) and his robotic surgical team. MISC was organized six months after the first RARP, and this department consisted of all the departments related to robotic surgery such as anesthesiology, urology, gynecology, respiratory surgery, digestive surgery, operation room nurses and medical engineers. From the view of safe implementation of robotic surgery, each surgery was performed under the supervision of the MISC. For instance, a certificate for surgery type and the console surgeon were authorized by MISC. Specifically, the MISC has a “termination order” authority, which is applied when there is excessive bleeding or surgical time [Figure 1]. Robotic surgery must be changed into other types of surgery such as open conversion once the order is given. Each robotic surgery case in four surgery departments is checked and discussed pre- and post-operatively in the regular meeting held by MISC twice a month. MISC renovated a wide operating theater for only robotic surgery, and this theater was separate from the conventional operating theaters [Figure 2].

### Data collection

Pre-, intra and post-RARP data collection of 100 cases was performed in prospective manner. The median (range) follow-up period was 11.9 (1.3 - 27.3) mon**t**hs. No patients were lost during follow-up. The collected data were categorized as follows: 1) pre-operative characteristics, 2) peri-operative data, 3) peri-operative complications, 4) pathological findings, 5) oncological outcome, 6) urinary function outcome, and 7) sexual function outcome. Biochemical recurrence (BCR) was defined as when the serum PSA level exceeded 0.2 ng/ml or when a radical prostatectomy was carried out and the PSA did not decrease below 0.2 ng/ml after surgery. The evaluation of urinary function outcome was performed using the International Prostate Symptom Score (IPSS) and number of daily pad use, and that of sexual function outcome using the International Index of Erectile Function (IIEF)-erectile function (EF) domain score (questions 1–5 and 15). The data of urinary and sexual function outcome were collected using questionnaires. The detail of the study was explained to each of patient, and written informed consent was received from all the patients. This study was approved by the institutional review board at Tottori University Faculty of Medicine and conducted in accordance with Declaration of Helsinki.

### Statistics

The comparisons of IPSS and IIEF-EF domain scores among different groups at the same time points were analyzed using the Mann–Whitney U test. The comparisons of those scores at different time points in one group were analyzed using the Wilcoxon signed-rank and Friedman tests.

## Results

### Case number results in the MISC

The case number results in the MISC as of December 31, 2012 are shown in Table 1. Each surgery type in Table 1 had been certified by the MISC. The certified committee surgeons included two from urology, one from gynecology, one from respiratory surgery and two from the digestive surgery departments.

**Table 1:** Actual results of whole robotic surgeries in MISC

	**Operative methods**	**No. cases**	**Expense claim**	**No. open conversion**
•Urology	*RARP	100	Medical insurance	0
	*Partial nephrectomy	12	Private expense	0
•Gynecology	*Hysterectomy	20	Institutional fund	0
•Respiratory surgery	*Lobectomy	18	Private expense	0
	*Thymectomy	14	Private expense	0
	*Posterior mediastinal tumor resection	1	Institutional fund	0
•Digestive surgery	*Gastrectomy	15	Institutional fund	1
	*Low anterior resection	8	Institutional fund	0
	Total	188		1

### Application of medical insurance for RARP

The use of any type of medical insurance was not applicable from October in 2010 until August in 2011. For insurance purpose, highly advanced medical technology procedures were applicable from August in 2011 until March in 2012. Japanese medical insurance has been applicable for RARP from April in 2012.

### Patients’ characteristics

The pre-operative patients’ characteristics are shown in Table 2. We defined the indication for RARP as localized prostate cancer; however, the National Comprehensive Cancer Network (NCCN) high-risk cases were considered as eligible in our policy after careful consideration in each case. In those cases, a wide resection of prostate together with extended lymphadenectomy was performed. Pre-operative assessment of PCa consisted of a digital rectal examination, radiological examinations including magnetic resonance imaging and computed tomography using contrast material, and trans-rectal ultrasound. The findings of these examinations were reflected in the pre-operative staging of PCa in each case. The staging of PCa was according to the International Union Against Cancer standards (Seventh Edition). Previous abdominal surgeries either open or laparoscopic were eligible for RARP in our policy.

**Table 2:** Pre-operative patients’ characteristics

**No. patients**	**100**
Continuous variables; mean (range)	
Age (ys)	64.6 (48 – 76)
BMI (kg/m^2^)	23.8 (18.0 – 35.4)
PSA (ng/ml)	9.5 (2.7 – 39.2)
No. clinical stage:	
T1c	27
T2a	38
T2b	6
T2c	20
T3a	9
No. Gleason score:	
6	27
7	38
8	23
9	11
10	1
No. IPSS:	
0 – 7	53
8 – 19	34
20 - 35	13

### Surgical procedure

Although surgical procedures were performed using a transperitoneal, 6-port technique based as previously described [7], modifications that considered aspects of our anatomical research were employed [8]. The anastomosis was performed as described by Van Velthoven et al. [9] with modifications. Nerve sparing procedures were performed using a similar method according to the four grades of postero-lateral resection of the prostate [10]: Grade 1, intrafascial dissection; Grade 2, interfascial dissection; Grade 3, extrafascial dissection (partial nerve sparing), and Grade 4, wide dissection.

### Operating time transition

The procedures in first 32 cases were performed by a single surgeon (A.T.) with considerable experience in open (ORP) and laparoscopic radical prostatectomy (LRP). Subsequently, a second pair (2 surgeons) of certificated surgeons entered into performing console procedures using step-by-step methods under the supervision of A.T. From the case number 71, another two certificated surgeons also entered into these procedures. The second and third pairs of certificated surgeons only had experience with ORP but not with LRP. The mean (range) total operative and console time except for the cases of extended lymphadenectomy in single surgeon (A.T.), the first pair and the second pair of his trainee doctors were 331 (225–575), 334 (251–409), 354 (307–399) and 244 (160–479), 249 (168–318), 273 (215–319) minutes respectively.

### Peri- and post-operative complications

There were no cases that required a conversion to open surgery, or intra-operative complications which required additional surgical procedures and intra-operative blood transfusions. Post-operative major complications included one patient who had bleeding from the neurovascular bundle (Clavien IIIa) and one patient had an hematoma located in abdominal muscle (Clavien II). Post-operative minor complications included eight patients with subcutaneous emphysema, five with anastomotic leaks, four with subcutaneous hemorrhages, two with ileus, two with lymphocele and seven cases with other complications. All minor complications were categorized as Clavien I.

### Oncological outcomes

Post-operative pathological characteristics are shown in Table 3. The positive surgical margin (PSM) rate defined by the presence of cancer cells at the inked margin was 19% for the entire series and was 10.7% for pT2, 42.9% for pT3a and 50% for pT3b disease. Additionally, 57.9% of PSM cases had extra-capsular extension disease. Biochemical recurrence (BCR) was observed in 9% of the entire patient series, and four patients (44.4%) had both a Gleason score 9 and lymph node metastases.

**Table 3:** Post-operative pathology

**No. patients**	**100**
No. pathological stage:	
T2a	15
T2b	5
T2c	55
T3a	21
T3b	4
No. Gleason score:	
6	11
7	64
8	13
9	12
No. Positive lymph nodes	5
No. PSM by stage (%):	
pT2	8/75 (10.7%)
pT3a	9/21 (42.9%)
pT3b	2/4 (50%)
All stage	19/100 (19%)
No. PSM location	
Apical	14
Lateral	1
Posterior	1
Anterior	1
Multifocal	2

### Pre- and post-operative sequential transitions of urinary function

**Figure 3:**
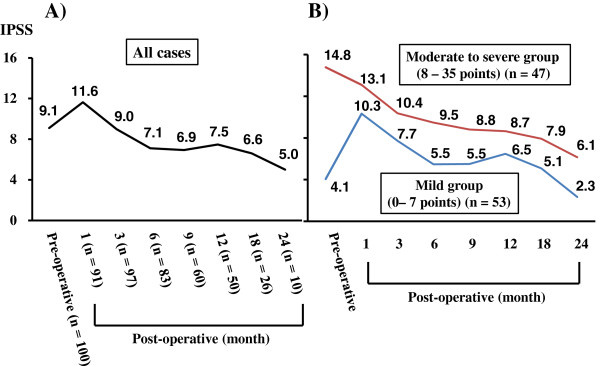
**The sequential transitions of pre- and post-operative IPSS were shown. A)** Total cases, and **B)** Patients divided into two groups according to the severity of pre-operative IPSS; Moderate to severe group, 8–35 points; Mild group, 0–7 points.

**Figure 4:**
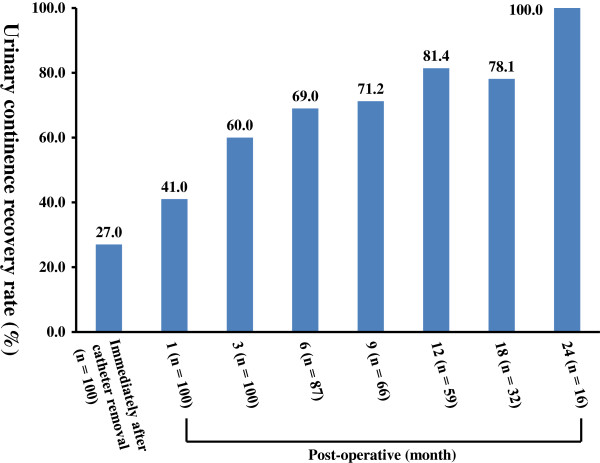
The post-operative continence recovery rates at precise time points are shown.

The sequential transitions of pre- and post-operative IPSS are shown in Figure 3. One month post-operative IPSS were significantly higher than pre-operative values (*P* = 0.0011); however, significant improvements were observed three months post-operatively (*P* < 0.0001), and improvements continued until 24 months post-operation [Figure 3A]. When the patients were divided into two groups based on the severity of pre-operative IPSS, contrasting transition values were seen between the moderate to severe (8–35 points) and mild (0–7 points) groups [Figure 3B]. Specifically, consistent improvements were observed in the moderate to severe group throughout follow-up duration (pre- vs. three months post operation; *P* = 0.0005). In contrast, one month post-operative IPSS were significantly higher than pre-operative values (*P* < 0.0001); however, significant improvements were observed three months post operation (*P* = 0.0001) in the mild group. With regard to urinary continence evaluation, post-operative continence was defined as no use of pad per day. The continence recovery rates immediately after catheter removal, 1, 3, 6, 9, 12, 18 and 24 months post operation were 27, 41, 60, 69, 71.2, 81.4, 78.1 and 100%, respectively [Figure 4].

### The analysis of potency recovery according to the grade of neurovascular preservation

**Figure 5:**
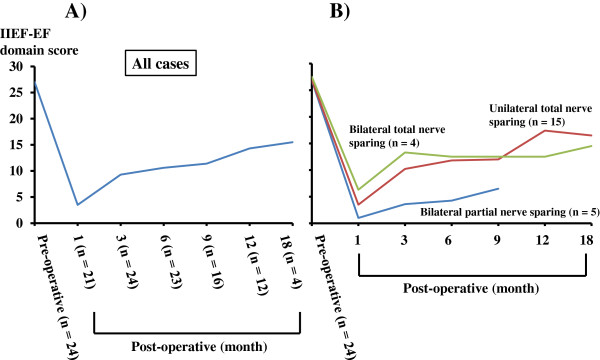
**The sequential transitions of pre- and post-operative IIEF-EF domain scores in patients with no and mild erectile dysfunction before surgery are shown. A)** Total cases, and **B)** Patients divided into three groups according to the status of neurovascular sparing procedures.

Potency recovery in the patients with no (IIEF-EF domain score; 26 – 30) and mild (IIEF-EF domain score; 22 – 25) erectile dysfunction before surgery were analyzed sequentially [Figure 5A]. Because of limited patient numbers, grades 1 and 2 were included in the same category of total nerve sparing. Grades 3 and 4 were categorized as partial and non-nerve sparing, respectively. In the analysis of bilateral- and unilateral total, and bilateral partial nerve sparing groups, these three groups demonstrated similar transitions; a sharp decrease of EF domain scores one month post-operation and improvements thereafter [Figure 5B]. The three month post-operative scores were significantly higher than the one month post-operative scores in the unilateral total nerve sparing group (*P* = 0.005). In comparison of scores among the three groups, bilateral- and unilateral total nerve sparing groups revealed higher scores than those of the partial nerve sparing group post-operation for all time points. This characteristic is significant at the one (bilateral total vs. bilateral partial; *P* = 0.0472) and six (unilateral total vs. bilateral partial; *P* = 0.0451) month post-operative time points.

## Discussion

Compared with the conventional laparoscopic technique, RARP provides the advantages including increased degrees of freedom and maneuverability, a three dimensional (3D) view with magnification, and a filtered tremor [11]. Apart from the innovative improvements of surgical techniques, the robotic program requires team-based training not only for surgeons but also for other medical staff who are involved in robotic surgeries. Steers et al. emphasized that the creation of a dedicated team of surgeons and nurses is necessary for the successful implementation of robotic surgery [12]. To accommodate the robot, the operation theater required renovation. To the best of our knowledge, the MISC is the first comprehensive organizational institution that runs robotic surgeries and spans over four different surgical departments. Moreover, the MISC is a renovated operating theater that covers a large area and has a sufficient numbers of wide 3D monitors for viewing by assistant surgeons and other observers. One notable issue, other than surgical skills and its correlation to patient outcome, is how to run a robotic surgery effectively from the aspects of cost, education and safety. Simple modifications in operating room processes were suggested to be associated with reduced costs and time for RARP [13]. Although large amounts of funding were necessary to begin the MISC, we believe that the cost-efficiency and educational benefit of running a robotic surgeries theater for MISC will improve in the near future.

One surgeon (A.T.) of our RARP team had sufficient experience in ORP and LRP, and successfully demonstrated a learning curve over the first 10 to 20 cases (data not shown in this article). This result is in accordance with the previous study in which the learning curve of an experienced open yet naïve laparoscopic surgeon demonstrating 4-hours surgical proficiency was 12 cases [14]. Because RARP is a team-based surgery, the assistant’s skills might have influenced the learning curve of our team. The introduction of 3D monitors for first assistant from case No. 9 might have contributed to the successful learning curve of our team because the first assistant had no experience of LRP. In contrast, if the learning curve was linked to the improvement of positive surgical margin rate, it was demonstrated that this started to plateau after 1000–1500 cases [15]. Because the new pentafecta concept is considered to be the recent true goal of RARP, the learning curve should not be discussed only with regard to the issue of operating time.

The following paragraphs discuss the issue of pentafecta. 1) Peri-operative complications: Our study demonstrated no cases in which intra-operative open conversion was required. A previous study reported seven cases of open conversion in their initial 100 cases of RARP, and five of those cases were due to surgical procedures problems [16]. The complication rate according to Clavien classification of our study is comparable with that of a previous study of 200 initial cases reported by Jeong et al.; however, the rate of severe complications other than Clavien I was much less in our study when compared with that report [17]. The structured program for the certification of console surgeons in the MISC and our original step-by-step training program referenced by a previous study [18] has been suggested to minimize the severe complications and the requirement of open conversion operations. 2) BCR and PSM rate: Because the follow-up duration is relatively short in our patient cohort, oncological control was only been evaluated by PSM rate to date. The entire PSM rate of 19% in our study is compatible to those of previous studies in initial patient cohort of 50–200 cases which demonstrated PSM rates of 30 [19], 16 [16], 23 [20] and 10.5 [7] %, respectively. More than half of our patient cohort did MRI examinations before the prostate biopsy. This pre-operative examination procedure enabled us to accurately detect cancer foci, and consequently led to an appropriate surgical policy with an excision range. We believe that an appropriate surgical policy and precise intra-operative procedures will contribute to lower the PSM rate as our experience increases. 3) Urinary function outcome: The natural history of urinary functional transition after RARP is currently poorly described. Our results demonstrate significant improvement of IPSS 3, 6, 12 months after RARP compared with that at pre-operative base-line in moderate to severe IPSS cases are accordance with the previous study [21]. This post-operative trend of IPSS improvement in moderate to severe IPSS cases was also demonstrated in ORP including retropubic [22] and perineal [23] approaches. As mentioned for ORP, RARP is suggested to also have a significant beneficial effect on lower urinary tract symptom (LUTS) beyond cancer control. Our results of continence recovery are compatible with the a recent review of a meta-analysis that demonstrated the 12-month urinary incontinence rates ranged from 4% to 31%, with a mean value of 16% not using a defined pad [24]. They concluded that the prevalence of post-operative continence is influenced by various factors including preoperative patient characteristics, surgeon’s experience, surgical technique, and methods used to collect and report data [24]. 4) Potency: The transition of post-operative potency recovery rate in patients treated by nerve sparing procedures were assessed at almost similar post-operative time points of 3, 6, and 12 months [6,25-27]. These studies demonstrated the effect of bilateral nerve sparing when using the definitions of either the Sexual Health Inventory for Men (SHIM) or an erection sufficient for intercourse. Although our data number are not sufficient to indicate significant conclusions, this is the first report to include additional detailed data with regard to more precise post-operative time points and according to three different types of nerve sparing procedures. We are continuing to collect data regarding these issues, and we believe that the definitive conclusions of precise post-operative transitions according to various types of nerve sparing procedures (three grades of preservation multiplying bi- or unilaterally) will be generated in the near future.

## Conclusions

This is the first report of an initial 100 RARP cases implemented in the constitutional framework of an academic institution. The MISC are providing immeasurable benefits from the aspects of patient safety and education for the robotic surgical team. RARP is a safe and efficient method for achieving PCa control along with functional preservation even during the initial experience for this procedure.

## Abbreviations

PCa: Prostate cancer; US: United States; RARP: Robot-assisted radical prostatectomy; MISC: Minimal invasive surgery center; PSM: Positive surgical margin; BCR: Biochemical recurrence; IPSS: International prostate symptom score; IIEF: International index of erectile function; EF: Erectile function; ORP: Open radical prostatectomy; LRP: Laparoscopic radical prostatectomy; 3D: Three dimensional.

## Competing interests

The authors declare that they have no competing interests.

## Authors’ contributions

TS, TM, SM, KH, NK, AY, KM and MH participated in concept, design, data collection, data analysis, data interpretation. TS participated in conccept, design, data collection, data analysis, data interpretation and writing. HK and AT made supervision of the study. All authors read and approved the final manuscript.
